# Tracking of the biochemical changes upon pleomorphic adenoma progression using vibrational microspectroscopy

**DOI:** 10.1038/s41598-021-97377-2

**Published:** 2021-09-09

**Authors:** Czesława Paluszkiewicz, Maciej Roman, Natalia Piergies, Ewa Pięta, Monika Woźniak, Mariangela Cestelli Guidi, Katarzyna Miśkiewicz-Orczyk, Magdalena Marków, Wojciech Ścierski, Maciej Misiołek, Bogna Drozdzowska, Wojciech M. Kwiatek

**Affiliations:** 1 Institute of Nuclear Physics, Polish Academy of Sciences, Radzikowskiego 152, 31-342 Kraków, Poland; 2 INFN-Laboratori Nazionali di Frascati, Via E. Fermi 40, 00044 Frascati, Italy; 3 Department of Otorhinolaryngology and Laryngological Oncology in Zabrze, Medical University of Silesia Katowice, 41800 Zabrze, Poland; 4 Department of Pathomorphology Zabrze, Medical University of Silesia, Katowice, Poland

**Keywords:** Lipids, Proteins, Head and neck cancer

## Abstract

Head and neck tumors can be very challenging to treat because of the risk of problems or complications after surgery. Therefore, prompt and accurate diagnosis is extremely important to drive appropriate treatment decisions, which may reduce the chance of recurrence. This paper presents the original research exploring the feasibility of Fourier transform infrared (FT-IR) and Raman spectroscopy (RS) methods to investigate biochemical alterations upon the development of the pleomorphic adenoma. Principal component analysis (PCA) was used for a detailed assessment of the observed changes and to determine the spectroscopic basis for salivary gland neoplastic pathogenesis. It is implied that within the healthy margin, as opposed to the tumoral tissue, there are parts that differ significantly in lipid content. This observation shed new light on the crucial role of lipids in tissue physiology and tumorigenesis. Thus, a novel approach that eliminates the influence of lipids on the elucidation of biochemical changes is proposed. The performed analysis suggests that the highly heterogeneous healthy margin contains more unsaturated triacylglycerols, while the tumoral section is rich in proteins. The difference in protein content was also observed for these two tissue types, i.e. the healthy tissue possesses more proteins in the anti-parallel β-sheet conformation, whereas the tumoral tissue is dominated by proteins rich in unordered random coils. Furthermore, the pathogenic tissue shows a higher content of carbohydrates and reveals noticeable differences in nucleic acid content. Finally, FT-IR and Raman spectroscopy methods were proposed as very promising methods in the discrimination of tumoral and healthy tissues of the salivary gland.

## Introduction

Salivary gland tumors are considered low-grade malignancies since approximately 80% of all cases are diagnosed as benign^1–3^. However, a strong predicting capacity for recurrence and a real risk of malignant form development determine an urgent therapeutic approach^1,2^. A crucial part of the salivary gland neoplasm diagnosis is the assessment of the glands abnormality. It is not only important for the classification of tissue as benign hyperplasia or malignant tumor but also in the case of tissue classification as benign type lesions^4^. This characterization results in a proper surgical perspective. Typically, for benign neoplasms, the lesion is removed with a margin of healthy tissue. In the case of high grade malignant tumors, treatment is radical and consists of the complete removal of the gland, in some cases even with the nerve excision^2^. The fine-needle aspiration (FNA) biopsy is a commonly used diagnostic tool for classification of the salivary gland tumors^4,5^. This fast method ensures cellular material for microscopic discrimination in a minimally invasive way providing relatively low discomfort for the patients. However, the appropriate distinction of the received material is strongly dependent on the experience of a cytopathologist^5–7^. It should be noted that there is a group of patients with benign tumors who do not decide to undergo surgical treatment due to the above-mentioned possible facial nerve paralysis. In such cases, the diagnosis is based only on the FNA biopsy. Nevertheless, some literature data is proving that such a diagnostic approach does not provide one hundred percent accuracy in distinguishing salivary gland tumors. Borsetto et al.^8^ showed data for 496 patients who were diagnosed with Warthin's tumor in the preoperative examination. In the postoperative examination for 4% of them, the malignant lesion was recognized. Naz et al.^9^ indicated that the sensitivity of FNA biopsy is about 85%. The authors pay attention to the lower diagnostic value of FNA in mixed tumors and recommend immunohistochemistry as the second stage of diagnosis in this case. The cited results confirm that, due to the occurrence of the various subtypes of the salivary gland tumors^10^ and their high histological complexity, the preoperative and intraoperative diagnosis remains an ongoing challenge^11,12^. Development of the current routine procedures used in cancer therapies by methods that could be helpful in the fast and effective diagnosis is still highly desirable.

The transformation of healthy cells into tumor cells is associated with the appearing changes in the molecular composition of lipids, proteins, and DNA^13,14^. These alterations can be tracked by the vibrational spectroscopies, namely Fourier transform infrared (FT-IR) and Raman spectroscopy (RS). Although both methods are based on different physical phenomena, they ensure complementary information about the molecular structure of the investigated species^15–17^. They are also able to capture any disruptions of this molecular construction involved in the disease expansion^18–20^. The main advantages of introducing FT-IR and RS in cancer diagnostics are undoubtedly the possibility to image a whole tissue without any labeling^21,22^ and avoid difficulties in histopathological interpretation^11^. On the other hand, the detailed characterization and focusing on the biochemical profile of the tumor in relation to the corresponding physiological tissue open up new possibilities for understanding the etiology of the disease development. This is extremely important in terms of selecting appropriate treatment and designing new strategies in the fight against cancer. Since disease diagnostics using vibrational spectroscopy is a rapidly growing field, the use of this method in diagnosing various types of cancer has been recently considered as a novel universal clinical method^23,24^. FT-IR and RS have been already applied for the chemical characterization of breast^25–29^, colon^30^, prostate^31–34^ lung^35^, brain^36^, skin^37^, and oral^38–42^ cancers with promising results. A similar strategy for the neoplasms of the salivary glands was examined^43–49^. An observed vanish for the FT-IR band attributed to the ester group vibrations (C=O) of triacylglycerols at 1745 cm^−1^^43^, typically strongly enhanced in the corresponding spectra of the physiological salivary gland tissue, was recognized as the main spectroscopic marker characteristic for this type of lesion. This observation was confirmed by the histological images^43^ which showed that the crucial transformation promoting the salivary glands' neoplasm development is associated with the changes in the content of adipose cells (fat cells) which is low in the malignant tissue. Another important process appearing within the tumor tissues was detected by comparing the relative intensity between the two FT-IR bands at ~ 1458 cm^−1^ (methyl bending vibrations) and ~ 1398 cm^−1^ (methylene bending/carboxylate stretching modes)^45^. The higher intensity of the latter band is a typical profile representing the participation of the hypomethylation along with tumorigenesis. Raman spectral patterns noticed for the salivary glands tumor tissues in relation to the appropriate margins also reveal significant differences. Yan et al.^49^ remarked the increased intensity of the particular bands attributed to the DNA/RNA and proteins suggesting high content and composition changes of these components within the considered tumor tissue. Moreover, the authors also observed the lipid content differentiation in physiological and tumor salivary gland tissues. Simultaneously, Yang et al.^48^ drew similar conclusions supported by statistical analysis. The main distinguished variations appearing upon the development of the neoplasm were reflected in structural changes in nucleic acids, proteins, collagen, lipids, and DNA/RNA.

Previously, nano-infrared spectroscopy investigations of pleomorphic adenoma, the most common tumor of the salivary glands, have been performed^50^. The obtained results reveal the significant changes in the protein secondary structure upon the disease development. Briefly, the protein fibrillization occurring within this type of neoplasm has been proved and visualized with nanoscale spatial resolution. In this work, the data obtained by the FT-IR and RS analysis of the pleomorphic adenoma in relation to the marginal tissues, have been discussed. The physiological salivary glands are strongly heterogeneous as they are rich in fat cells that aggregate and their distribution within the tissue is not uniform. Therefore, the application of the methods with quite high spatial resolution may ensure misleading interpretation which is strongly connected with the investigated area of the sample. The deep characterization using FT-IR and RS, which provides different spatial resolutions (in this study ~ 2.7 μm and ~ 0.5 μm, respectively), allows for a better understanding of the influence of experimental approaches on the obtained information. Additionally, to capture subtle changes in the obtained spectral data, the principal component analysis (PCA) supports the performed considerations. The applied experimental and statistical approaches ensured possibilities to recognize any subtle biochemical changes in the investigated tumor tissues in relation to the adequate margins. These changes are reflected mainly in the lipid content. However, it appears that also other biomolecular components influence this tumor progression. Although the pleomorphic adenoma tumor is well-circumscribed and the distinguishing between this tissue and the margin should not pose any major problems^51^, the deep elucidation of the chemical changes appearing upon disease development seems to be crucial. Such an approach ensures valuable information about the mechanisms responsible for tumor progression. On the other hand, the detailed spectral characterization of the biochemical content and conformation changes characteristic for the particular tumors (in comparison with the margins) may allow to improve and support the current diagnostic procedures^52–55^.

## Results and discussion

**Figure 1:**
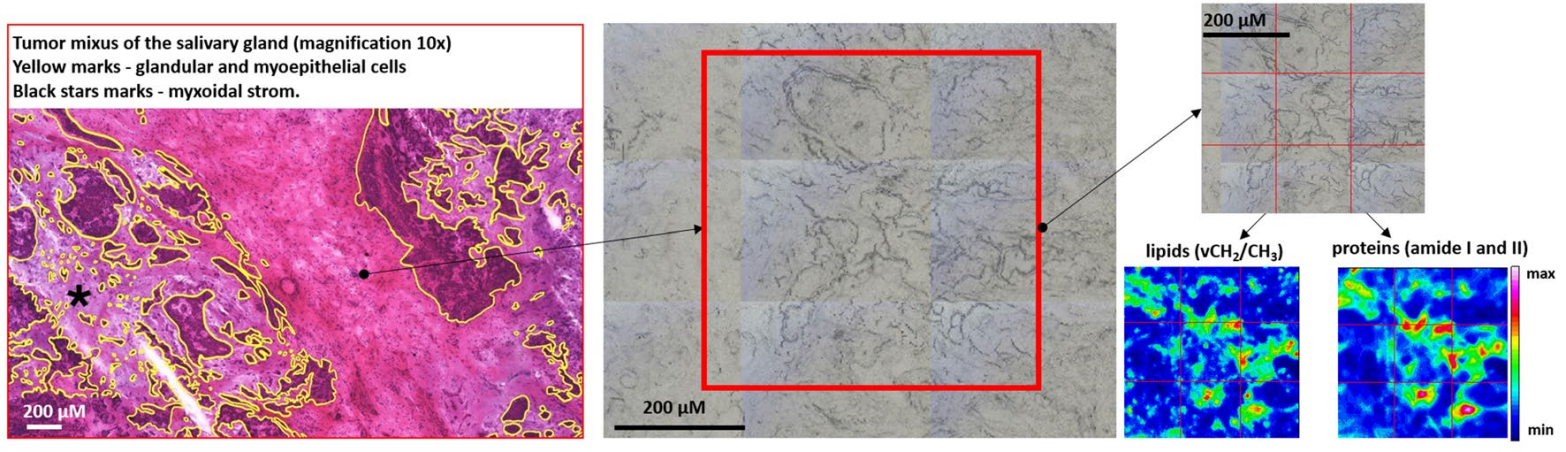
Exemplary microphotographs of hematoxylin and eosin (H&E) stained tumoral tissue biopsies, FT-IR microscope images, and FT-IR chemical maps distribution of lipids and proteins.

**Figure 2:**
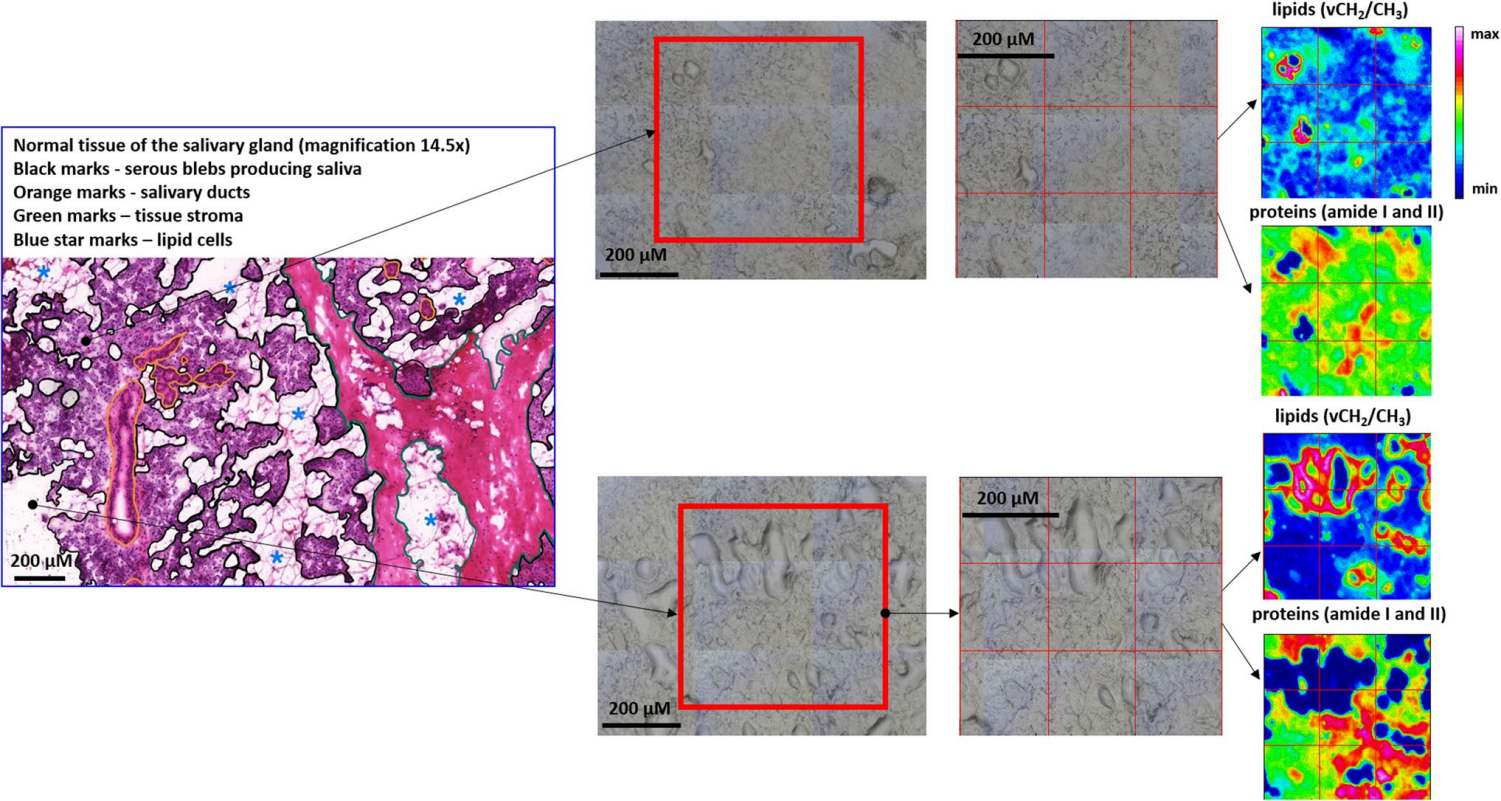
Exemplary microphotographs of hematoxylin and eosin (H&E) stained tissue biopsies of healthy margin, FT-IR microscope images, and FT-IR chemical maps distribution of lipids and proteins.

Figure 1 displays an exemplary microphotograph of hematoxylin and eosin (H&E) stained tumoral tissue biopsies with FT-IR microscope images as well as chemical distribution maps of lipids (CH_2_ and CH_3_ stretching vibrations) and proteins (Amide I oscillations). Consequently, Fig. 2 shows microphotographs of H&E stained tissue biopsies of healthy margin together with spectroscopic results. Histopathological analysis of the stroma and parenchyma confirmed the neoplastic changes in salivary tumoral gland tissues and were classified as tumor mixtus. The pleomorphic adenoma (tumor mixtus) consists of mixed epithelial and myoepithelial cell components in a chaotic arrangement. The performed histological staining confirmed the morphological changes between salivary gland tumor and non-pathological margin. The numerous nuclei aggregations with irregular shapes are visible in the pathological tissue in Fig. 1. Furthermore, it was noticed that within the healthy part there are areas that differ greatly in lipid content, as shown in the accompanying distribution maps (Fig. 2). These findings were the starting point for further analysis of the studied tissues and shed new light on the key role of lipids in comparative analysis.

**Figure 3:**
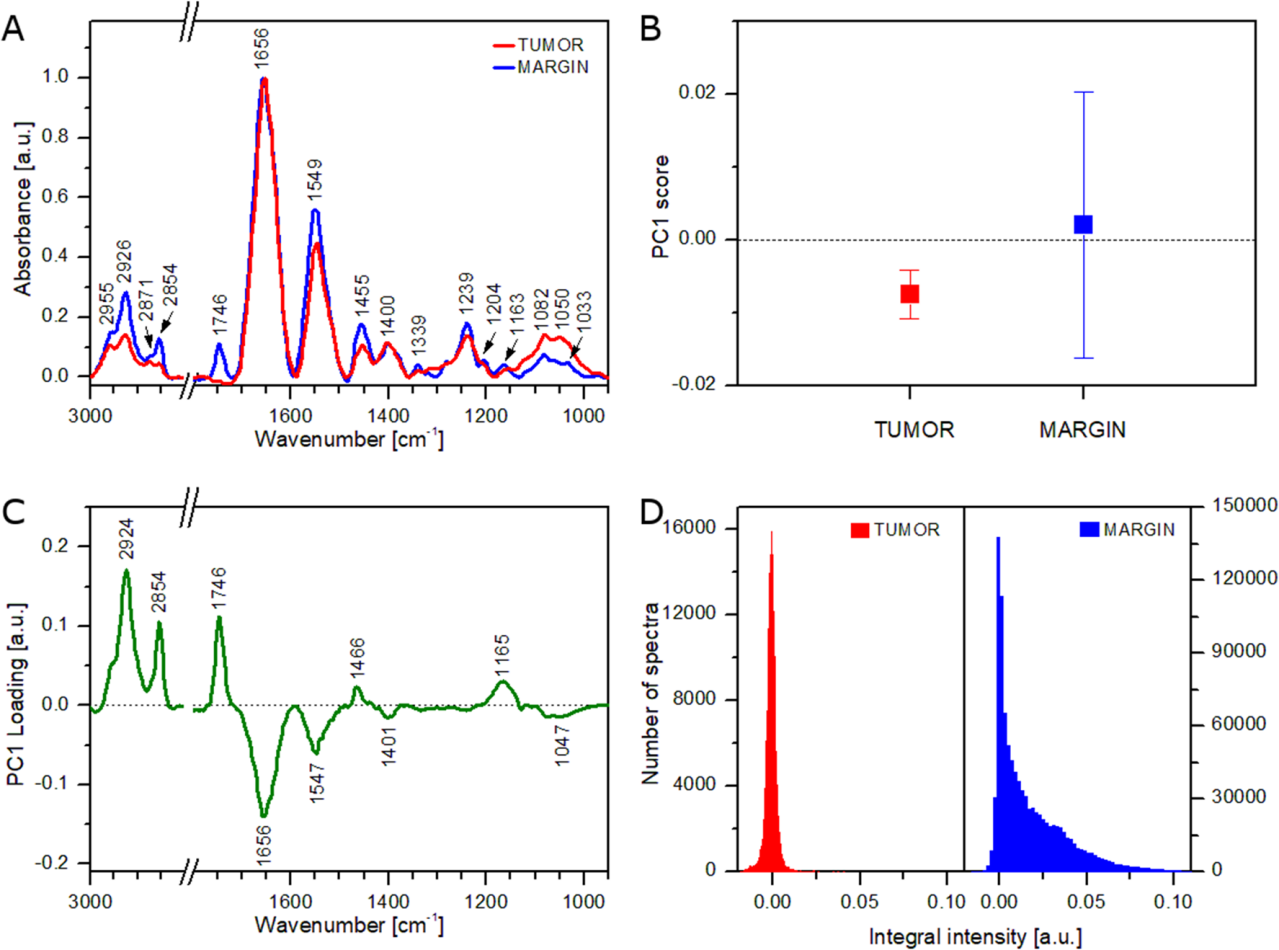
Results of FT-IR analysis of tumoral tissue and healthy margin using all acquired spectra: average FT-IR spectra (**A**), mean values of PC1 score (**B**), PC1 loading plot (**C**), histograms showing the distribution of integral intensity of the 1746 cm^−1^ band (**D**).

FT-IR spectra collected from the healthy margin and tumoral tissue were averaged and compared to analyze the overall difference between both parts (Fig. 3A). Although both spectra show a similar pattern, a distinct difference can be noticed in the intensities of some bands. For instance, the FT-IR spectrum of tumoral tissue shows a higher relative intensity of the carbohydrate and nucleic acid bands (1000–1100 cm^−1^), whereas the spectrum of the healthy margin exhibits a higher relative intensity of some lipid bands (1455, 1746, 2800–3000 cm^−1^). However, due to the high heterogeneity of the gland tissue (Figs. 1, 2), the use of average spectra for differentiation between tumoral and healthy parts seems to be questionable. Thus, PCA analysis was applied for all acquired FT-IR spectra (full dataset) to find spectral features responsible for the discrimination of both types of tissue. Figure 3B presents mean PC1 score values with standard deviations (SDs) for the tumoral tissue and healthy margin. As can be seen, the tumoral tissue exhibits a negative value of PC1 score, whereas the healthy margin shows a positive value. Spectral features related to the PC1 are represented by a PC1 loading plot (Fig. 3C). The plot shows positive features at 1165, 1466, 1746, 2854, and 2924 cm^−1^. These signals can be assigned to lipids indicating the healthy margin to be richer in lipid domains than the tumoral tissue^56^. Contrarily, the tumoral tissue exhibits higher content of carbohydrates and proteins as negative features in the PC1 loading plot can be found at 1047, 1547, and 1656 cm^−1^ (Fig. 3C). The results are in good agreement with already published data (see Refs.^43–45^). It is worth highlighting that an SD of the mean PC1 score value of the healthy margin is approximately 5 times higher than the corresponding SD value for the tumoral tissue (Fig. 3B). Furthermore, the SD of the healthy margin (ca. 68% of the PC1 values) completely covers the mean ± SD values of the tumoral tissue. To support these findings, tumor-margin classification was performed on the basis of the PC1 score values. The area under the receiver operating characteristic curve (AUC) was calculated to reach the value of 0.60 that is only slightly better than 0.50 (no discrimination capacity). It means that the healthy margin is an extremely heterogeneous tissue with domains rich in lipids and areas of lipid content similar to the tumoral part. These results are in good accordance with FT-IR imaging observations (see Figs. 1, 2) and can be also illustrated by histograms showing the distribution of integral intensity of the 1746 cm^−1^ band (Fig. 3D). The histogram obtained for the tumoral tissue shows a normal distribution with a center close to 0.000. The highest intensity calculated for FT-IR spectra from this tissue was ca. 0.040. It proves that this tissue is homogeneous and poor in lipids. On the other hand, the histogram obtained for the healthy margin exhibits a right-skewed distribution with median and mean values close to 0.009 and 0.016, respectively. However, the maximum of the distribution curve is located at 0.000. The highest intensity calculated for FT-IR spectra from this tissue was ca. 0.110. It confirms high heterogeneity of lipid distribution in the healthy margin and the presence of lipid-rich domains across this part of a tissue.

**Figure 4:**
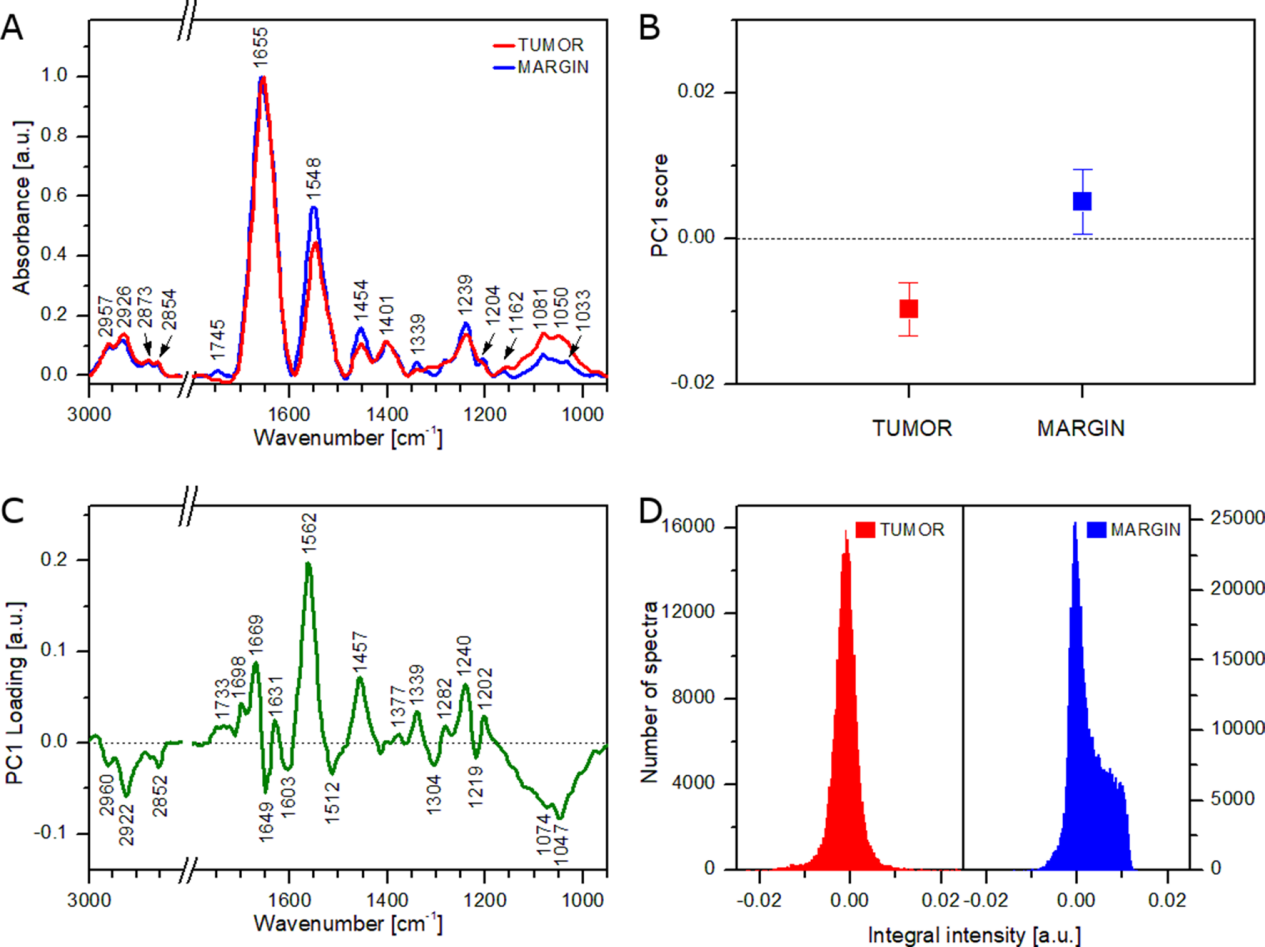
Results of FT-IR analysis of tumoral tissue and healthy margin without spectra showing lipid bands of high intensity: average FT-IR spectra (**A**), mean values of PC1 score (**B**), PC1 loading plot (**C**), histograms showing the distribution of integral intensity of the 1746 cm^−1^ band (**D**).

As lipid domains strongly influence the results obtained from both tissue regions, the analysis was repeated without FT-IR spectra showing lipid bands of high intensity. Figure 4A presents new average FT-IR spectra of the tumoral tissue and healthy margin. As can be seen, the spectrum obtained from the healthy margin does not exhibit strong lipid bands. Moreover, slight differences between both spectra can be observed for bands located at 1239, 1454, 1548 cm^−1^ and in the region of 1000–1150 cm^−1^. Since both tissues can be still heterogeneous to some extent, PCA analysis was performed for all FT-IR spectra of the reduced dataset (approx. 67% of the full dataset). Mean ± SDs values for PC1 score (Fig. 4B) indicate a good separation between the tumoral tissue and healthy margin. It was additionally confirmed by performing tumor-margin classification. The AUC value was found to increase up to 0.99 for the dataset with no lipid-rich spectra. It means that PCA analysis based on such a dataset provides almost perfect discrimination between both tissue types. As a consequence of eliminating lipid-rich spectra, the PC1 loading plot exhibits a completely different spectral pattern than the corresponding plot calculated for the full dataset (compare Figs. 3C, 4C). The current loading plot exhibits features related mainly to proteins and carbohydrates. For instance, a broad negative band with a maximum at 1047 cm^−1^ can be assigned to C–O stretching modes of carbohydrates^56,57^ and suggests a higher content of this type of biomolecules in the tumoral tissue. Moreover, the loading plot shows several bands typical for proteins such as Amide I (1631, 1649, 1669 cm^−1^), Amide II (1562 cm^−1^), Amide III (1240, 1282, 1304 cm^−1^), and other protein bands (1339, 1457, 2922, 2960 cm^−1^)^56,58^. The sign (positive, negative) of the corresponding FT-IR bands can be used for the estimation of protein secondary structure content^59^. A negative feature of the Amide I band observed at 1549 cm^−1^ suggests a higher content of proteins rich in unordered random coils and turns in the tumoral tissue^58^. On the other hand, a positive feature located at 1669 cm^−1^ suggests the healthy tissue contains more proteins in the anti-parallel β-sheet conformation^58^. As can be seen from the performed analysis, the effect of lipids on PCA results can be significantly reduced. However, this impact cannot be eliminated completely. Figure 4D presents histograms of the integral intensity of the 1746 cm^−1^ band calculated for both studied tissue types. Since no FT-IR spectra were removed from the dataset of the tumoral tissue (FT-IR spectra showed no lipid bands of high intensity), the histogram calculated for this type of tissue (left panel) shows the same structure as the histogram presented in Fig. 3D (left panel). Contrarily, the histogram calculated for the healthy margin exhibits a shoulder towards higher values of the integral intensity (Fig. 4D, right panel). The shoulder is cut off at ca. 0.014 due to the removal of the FT-IR spectra showing lipid bands of high intensity. As a consequence, median and mean values of the histogram calculated for the healthy margin are much closer to 0.000 (0.001 and 0.002, respectively), but still slightly higher than the corresponding values calculated for the tumoral tissue (0.000). It means that lipid contribution may further affect the spectroscopic analysis of salivary gland tissues, but such effect seems to be minor.

**Figure 5:**
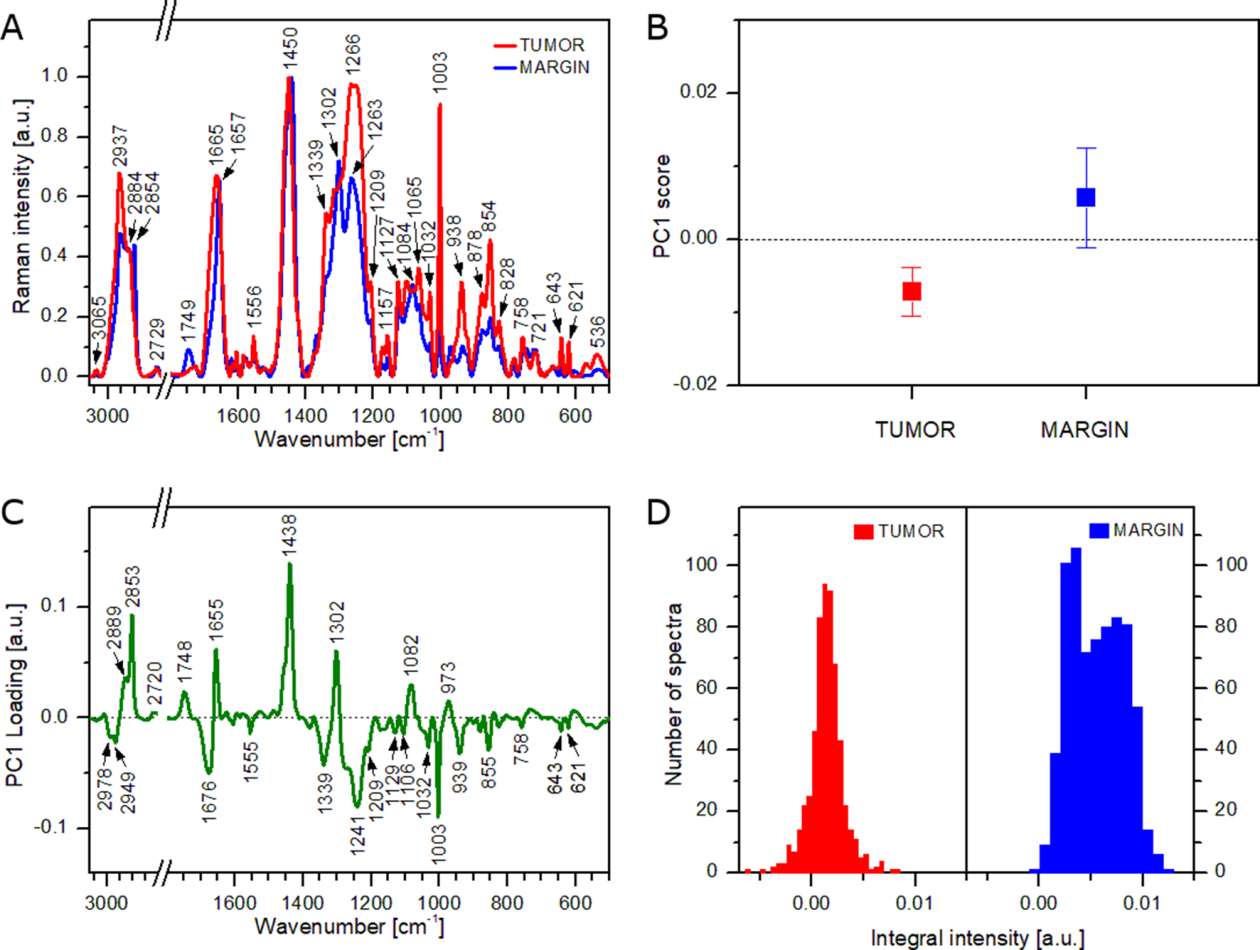
Results of Raman analysis of tumoral tissue and healthy margin using all acquired spectra: average Raman spectra (**A**), mean values of PC1 score (**B**), PC1 loading plot (**C**), histograms showing the distribution of integral intensity of the 1746 cm^−1^ band (**D**).

The studied tissue samples were also investigated using Raman spectroscopy, which is a complementary method to FT-IR. The analysis was carried out to highlight differences between the tissue types and compare results obtained by both methods. As performed for the FT-IR experiment, in the first step, Raman spectra acquired from both tissues (tumor and healthy margin) were averaged and contrasted (Fig. 5A). The average Raman spectra show clear differences in spectral pattern, especially in the range of 1200–1400 cm^−1^. However, a lot of other minor differences can be observed between both spectra. Thus, similarly to the analysis of FT-IR spectra, PCA was carried out using Raman spectral dataset (full dataset). Mean ± SDs values for PC1 score (Fig. 5B) suggest a good separation between the tumoral tissue and healthy margin. It was additionally confirmed by performing tumor-margin classification. The AUC value was calculated to equal 0.96. It proves that PCA analysis based on Raman spectral dataset shows a very high discrimination capacity for both tissue types. The corresponding PC1 loading plot is shown in Fig. 5C. All distinct positive features (973, 1082, 1302, 1438, 1655, 1748, 2853, and 2889 cm^−1^) observed in this plot can be assigned to lipids^56,60^. Moreover, positions of the bands suggest that unsaturated triacylglycerols are the main components of lipid domains. On the other hand, all clear negative signals (621, 643, 855, 939, 1003, 1032, 1129, 1209, 1241, 1339, 1676, 2949, and 2978 cm^−1^) come from vibrations of proteins^56,61^. Thus, it is obvious that PC1 differentiates the tumoral tissue from the healthy margin based on the proteins to lipids ratio. The same tendency was observed for FT-IR results obtained for the full dataset (see Fig. 3) and in the literature^46–49^. However, in this case, the capacity of the PC1-based discrimination between both types of tissue is much higher than observed for PCA performed using FT-IR spectra (compare Figs. 3B, 5B). The higher content of lipids in the healthy margin can be also illustrated by histograms showing the distribution of integral intensity of the 1746 cm^−1^ band (Fig. 5D). The histogram obtained for the tumoral tissue shows a normal distribution with a center at 0.001. Contrarily, the histogram calculated for the healthy margin exhibits two maxima located at 0.003 and 0.008. It confirms the heterogeneous composition of the margin tissue, which is rich in areas of lipid domains.

**Figure 6:**
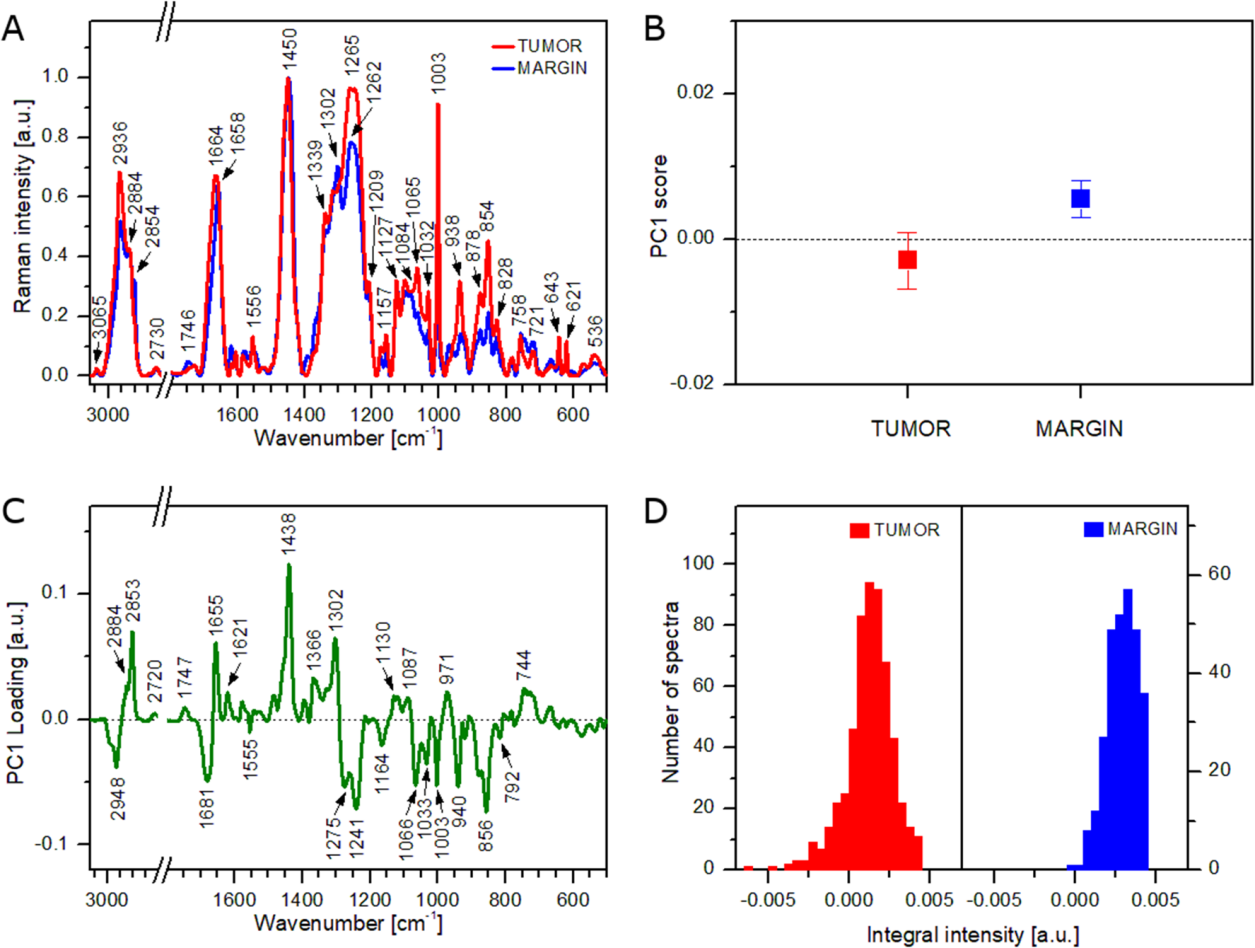
Results of Raman analysis of tumoral tissue and healthy margin without spectra showing lipid bands of high intensity: average Raman spectra (**A**), mean values of PC1 score (**B**), PC1 loading plot (**C**), histograms showing the distribution of integral intensity of the 1746 cm^−1^ band (**D**).

Because lipid domains strongly influence the results of Raman measurements, the analysis was repeated without spectra showing lipid bands of high intensity. Figure 6A presents new average Raman spectra of the tumoral tissue and healthy margin. As can be seen, lipid bands (e.g. 1084, 1302, 2854 cm^−1^), observed mainly in the average spectrum of the healthy margin, show much lower intensities than the same bands present in average spectra calculated for the full dataset (compare Figs. 5A and 6A). As previously, PCA was performed for the reduced dataset to check the discrimination capacity of the data and identify spectral features responsible for tissue differentiation. Figure 6B presents mean ± SDs values for PC1 score with good separation between both tissue types. The AUC value of the tumor-margin classification was found to exceed 0.96. The value is very close to that obtained for the full Raman dataset. Figure 6C presents the PC1 loading plot of the performed PCA. Most of the positive and negative features are located at similar wavenumbers as observed in the corresponding plot of PCA performed for all Raman spectra (compare Figs. 5C and 6C). These include positive signals at 971, 1087, 1302, 1438, 1655, 1747, 2853, and 2884 cm^−1^ originating from lipids as well as negative signals at 856, 940, 1003, 1033, 1241, 1681, and 2948 cm^−1^ coming from proteins. Additionally, the plot exhibits spectral features at 744 (DNA), 792 (DNA), 1066 (proteins and lipids), 1130 (proteins and lipids), 1164 (proteins), 1275 (proteins), 1366 (lipids), and 1621 cm^−1^ (C=C stretching vibrations). It seems obvious that discrimination between the tumoral and healthy margin is based on the lipids to proteins ratio. The healthy margin contains more lipids, whereas the tumoral tissue is richer in proteins. Moreover, a slight difference in DNA composition can be also observed between both tissue types. The higher lipid content of the healthy margin can be confirmed by histograms showing the distribution of integral intensity of the 1746 cm^−1^ band (Fig. 6D). The tumoral tissue shows a normal distribution with a center at 0.001 as obtained for the full dataset (compare Figs. 5D and 6D). Conversely, the histogram calculated for the healthy margin exhibits a maximum shifted up to 0.003.

Results obtained with both FT-IR and Raman spectroscopies revealed the usefulness of these methods in the discrimination of tumoral and healthy tissues of the salivary gland. Both methods exhibit high discrimination capacity (0.99 and 0.96 for FT-IR and RS, respectively) based on PCA. Spectral analysis of the studied tissue types revealed the crucial importance of lipid domains for successful discrimination. The results obtained for the full FT-IR dataset showed no discrimination capacity due to high heterogeneity (presence of areas rich and poor in lipid domains) of the tumoral tissue. However, almost perfect discrimination (AUC = 0.99) was achieved after the removal of all FT-IR spectra showing lipid bands of relatively high intensity. The PC1 loading plot calculated for the FT-IR dataset modified in such a way exhibited features related mainly to proteins and carbohydrates. On the contrary, the results obtained for Raman spectra indicated proteins to lipids ratio as the main discriminant between tumoral and healthy tissues, regardless of whether the analysis was performed with all spectra or after removal of lipid-rich ones. The difference between both methods is related to the nature of both phenomena as well as the specificity of instrumentation and measurement mode. Indeed, FT-IR and Raman spectroscopy differ in spatial sampling scale and chemical sensitivity. FT-IR imaging allows measurements of large and representative areas of tissue in a reasonable time, whereas Raman imaging is limited to smaller sample areas (see “Materials and methods” section and Ref.^33^ for more details). Furthermore, FT-IR is rich in information about proteins and carbohydrates, whereas Raman spectroscopy is very sensitive to lipids^62^. Although both used spectroscopic methods show different spectral features responsible for tumor-margin discrimination, the obtained results suggest the potential utility of vibrational spectroscopy in chemical imaging of salivary gland tissues. However, caution should be taken whenever interpretation is based only on results obtained from one of the methods.

The diagnosis of the salivary gland neoplasm is based on visual inspection of tissue and the assessment of its abnormality. Moreover, recent spectroscopic studies indicated lower lipid content as a main marker of the tumoral salivary gland tissue. Although our study confirmed this tendency, we noted that a healthy margin is a highly heterogeneous tissue at the micro level with areas rich (lipid domains) and poor in lipids. Extreme caution should be taken whenever healthy margin areas of low lipid content are investigated as these areas can be easily confused with the tumor. To avoid any incorrect tissue assignment, we encourage to analyze areas poor in lipids in detail. Our results showed that significant changes in nucleic acids, proteins, and carbohydrates appear upon the development of the neoplasm in the salivary gland tissue. Such changes seem to be a more reliable indicator of the neoplastic progress at the micro level. Thus, our considerations provide not only the distinguishing between pleomorphic adenoma and the marginal tissue sections but also enable a better understanding of the mechanism of tumor transformation. The obtained results proved the usefulness of vibrational spectroscopy in conjunction with PCA to track subtle changes and to indicate spectral markers of salivary gland tumor progression. Furthermore, our study showed how the choice of the method (FT-IR or RS) influences the results and their interpretation. FT-IR is very sensitive to changes in the content of proteins and carbohydrates, whereas even minor changes in lipid content can be easily followed by Raman spectroscopy. Finally, this study has shown the utility of both applied vibrational spectroscopy methods in the discrimination of tumoral and healthy tissues of the salivary gland with a discrimination capacity of 0.99 and 0.96 for FT-IR and RS, respectively.

## Materials and methods

### Tissue samples

In this study, we analyzed the structure and chemical composition of the selected head and neck tissues: tumoral and non-affected by pathological changes (margins) from ten middle-aged patients suffering from salivary gland tumor (tumor mixtus). The materials were delivered from the Department of Otorhinolaryngology and Laryngological Oncology, Medical University of Silesia in Zabrze. All research has been approved by the Ethics Committee of Medical University of Silesia (No. KNW/022/KB1/32/I/17). Informed consent was obtained from all patients for being included in the study. Fresh samples were obtained during the surgical excision of tumors and then all the materials were immediately cryo-sectioned into two groups of slides until the experiments begin. The first part of the slides was taken for histopathological staining by hematoxylin and eosin solutions. The interpretation of obtained images was reviewed by a specialized pathologist to verify neoplastic changes. The second batch of non-staining slide sections was subjected to comprehensive vibrational spectroscopic analysis. Two slide sections were analyzed for each patient and tissue type. It means that 40 tissue sections (2 sections × 2 tissue types × 10 patients) were analyzed using vibrational spectroscopy. Prior to spectroscopic measurements, the 10 µm slide sections from tumoral and healthy margin tissues were placed onto CaF_2_ windows. This study was performed under the ethical standards of the World Medical Association Declaration.

### Histopathological staining

As a method of tissue classification, we used standard histological staining. The below-described technique is a commonly used protocol in diagnostic laboratories. Firstly, the frozen samples were cut into 10 µm thick sections by cryostat and immediately fixed with 96% ethanol. Then, the dissected tissue was subjected to incubation in a 10% formalin solution for 24 h. Afterward, slides were gently rinsed with sterile water and stained with hematoxylin for 30 s, rinsed under running water for 5 min, and then counterstained with eosin Y for 15 s. Finally, the tissue was dehydrated in ethanol (96% and 99.8%), fixed with xylene, and coverslipped with a mounting medium. All the diagnostic performances were evaluated by the professional pathologist using a microscope.

### FT-IR study

For each tumoral and healthy margin tissue, four maps (530 μm × 530 μm, 2 sections × 2 maps) from different areas were collected. It means that 40 FT-IR maps (4 maps × 10 patients) were acquired from each type of tissue (80 FT-IR maps in total). Since the margin is strongly heterogeneous, for sections of this tissue two maps were collected separately for lipid-rich and lipid-poor regions. FT-IR maps of the studied tissues placed onto CaF_2_ windows were collected using a HYPERION 3000 microscope coupled with a Vertex 70v spectrometer (Bruker Optics, Ettlingen, Germany). The microscope was equipped with a 15 × magnification objective and a 64 × 64 pixels focal plane array (FPA) detector (2.66 μm projected pixel size). The spectral data were registered in transmission mode in the range from 3800 to 900 cm^−1^ with a spectral resolution of 4 cm^−1^. The number of scans per spectrum was 256.

### Raman spectroscopy study

Similarly to the FT-IR measurements, four Raman maps (15 µm × 15 µm, 2 sections × 2 maps) for each tumoral and healthy margin tissue were acquired (40 Raman maps from each type of tissue, 80 Raman maps in total) using a Renishaw inVia Raman spectrometer coupled with a confocal microscope with a 100 × magnification objective and a CCD camera (1024 × 256 pixels). A 785 nm diode laser was used as the excitation source with the power at the sample surface of ca. 23 mW. Spectral maps were registered from three different places of the tissue sample with ca. 1 cm^−1^ spectral resolution covering the interval from 500 to 3200 cm^−1^. The exposure time was 60 s, and one scan was enough to obtain a good signal-to-noise ratio. The spectrometer was calibrated using the Raman scattering line generated by an internal silicon plate. The same tissue sections placed onto CaF_2_ windows were measured by both FT-IR and RS methods to compare the results. The sample surface was controlled after each measurement to confirm the absence of laser radiation damage.

### Data analysis

Prior to analysis, FT-IR and Raman spectra were preprocessed to improve data quality. Firstly, low-intensity spectra were removed due to the low signal-to-noise ratio (SNR). Then, both FT-IR and Raman spectra were smoothed (Savitzky-Golay, 13 and 7 smoothing points, 5th and 3rd polynomial order for FT-IR and RS, respectively), baseline corrected (Rubberband algorithm), and normalized (‘area under the curve’ normalization). When necessary, a cosmic ray removal procedure was applied to Raman spectra. Then, PCA was performed on FT-IR and Raman datasets to determine chemical differences between the samples. PCA was also used to check for any variation among the patients (Figs. S1 and S2, Supplementary Information). The tumor-margin classification was performed on the basis of the PC1 score values. The predictive power of the models was calculated using the area under Receiver Operator Curve (AUC). Data processing, as well as PCA analysis, were performed with MATLAB R2018b software (MathWorks, Inc., USA) using built-in and home written routines.

## Supplementary Information

Supplementary Figures.

## Data Availability

The datasets generated during and/or analyzed during the current study are available from the corresponding author on reasonable request.
