# Supplementary material for: Tracking of the biochemical changes upon pleomorphic adenoma progression using vibrational microspectroscopy
Source: Sci Rep. 2021 Sep 9;11:18010. doi: 10.1038/s41598-021-97377-2 (PMC8429647; doi:10.1038/s41598-021-97377-2)
Supplement: Supplementary file 1 — Supplementary Figures. [file 41598_2021_97377_MOESM1_ESM.docx]

**SUPPLEMENTARY INFORMATION**

**Tracking of the biochemical changes upon pleomorphic adenoma progression using vibrational microspectroscopy**

Czesława Paluszkiewicz^1*#^, Maciej Roman^1*#^, Natalia Piergies^1#^, Ewa Pięta^1#^, Monika Woźniak^1^, Mariangela Cestelli Guidi^2^, Katarzyna Miśkiewicz-Orczyk^3^, Magdalena Marków^3^, Wojciech Ścierski^3^, Maciej Misiołek^3^, Bogna Drozdzowska^4^, Wojciech M. Kwiatek^1^

*^1^Institute of Nuclear Physics Polish Academy of Sciences, PL-31342 Krakow, Poland*

*^2^INFN - Laboratori Nazionali di Frascati, Via E. Fermi 40, 00044 Frascati, Italy*

*^3^Department of Otorhinolaryngology and Laryngological Oncology in Zabrze, Medical University of Silesia Katowice, PL-41800 Zabrze, Poland*

*^4^* *Department of Pathomorphology Zabrze, Medical University of Silesia Katowice, Poland*


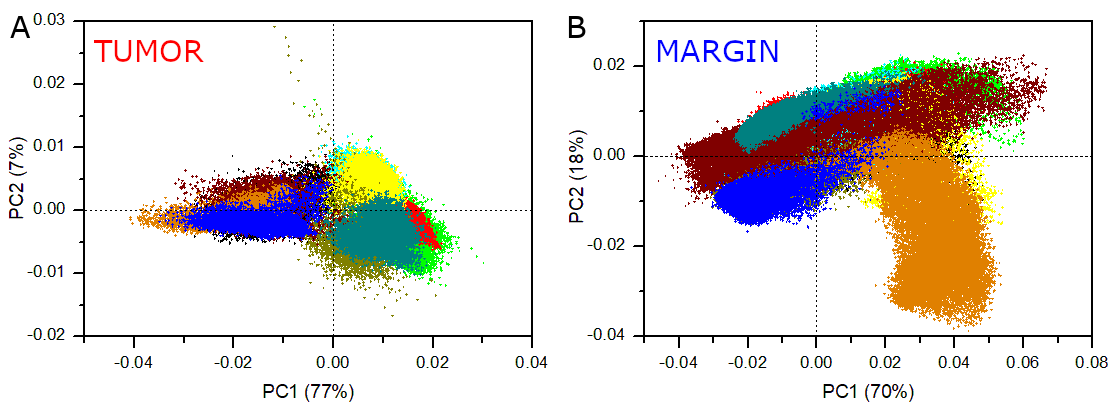


Fig. S1. Results of PCA performed on FT-IR spectra collected from various patients, and coming from: tumoral tissue (A) and healthy margin (B).


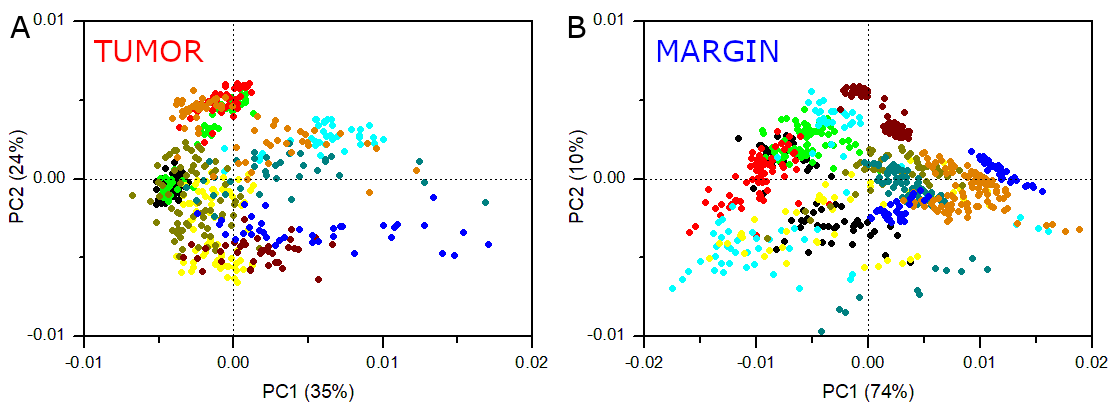


Fig. S2. Results of PCA performed on Raman spectra collected from various patients, and coming from: tumoral tissue (A) and healthy margin (B).

Figs S1 and S2 show results of PCA performed on FT-IR and Raman spectra collected from various patients, respectively. The presented scores plots for both tissue types, i.e. tumoral tissue and healthy margin, suggest no clear variation among the patients.
